# NextGenDO: A Pre-medical Outreach Program to Promote Early Exposure to Osteopathic Medicine

**DOI:** 10.7759/cureus.100341

**Published:** 2025-12-29

**Authors:** Kenneth A Quezada, Ledio Gjunkshi, Nadiya A Persaud, Robert Hasty

**Affiliations:** 1 Research, Orlando College of Osteopathic Medicine, Winter Garden, USA

**Keywords:** experiential learning, medical education, medical school education, osteopathic, osteopathic medicine, pre-medical outreach, pre-med student preparedness, student perceptions

## Abstract

The osteopathic profession continues to grow, yet awareness of its philosophy and training remains limited among students exploring medical careers. Early outreach initiatives can play an important role in improving exposure and garnering interest in the osteopathic pathway. The NextGenDO program was developed as a single-day, immersive event that introduced high school students to osteopathic medicine through gamified activities, hands-on learning, and interaction with medical students. This prospective, observational study evaluated the program’s impact using pre- and post-surveys. A total of 26 students were found eligible to participate, with most completing both surveys. Participants represented multiple grade levels and diverse backgrounds. Following the event, students demonstrated increased familiarity with osteopathic medicine, with survey analysis confirming a statistically significant improvement. Improvements were also noted in interest in medicine, confidence to pursue a medical career, and understanding of the pathway to becoming a physician, although these changes did not reach statistical significance. Post-event responses indicated that the majority of students were more likely to consider osteopathic medicine as a career, expressed interest in shadowing a physician, and were eager to attend similar outreach events. These findings support the value of early exposure programs in enhancing awareness of osteopathic medicine and encouraging interest in future medical careers.

## Introduction

In the United States, physicians are trained through two primary pathways: allopathic medicine, which awards the Doctor of Medicine (MD) degree, and osteopathic medicine, which awards the Doctor of Osteopathic Medicine (DO) degree. Osteopathic medicine is rooted in a philosophy that “focuses on treating the body as a unit with a natural tendency toward health and self-healing” [1]. Osteopathic physicians receive training in all aspects of modern medicine while also learning osteopathic manipulative treatment (OMT) as part of their approach to patient care. This dual emphasis on biomedical science and osteopathic principles offers a holistic, patient-centered framework that distinguishes the DO profession within the broader healthcare system.

The osteopathic profession has experienced substantial growth, with DO students comprising 28% of all US medical students during the 2023-2024 academic year [2]. As the number of osteopathic medical students continues to grow, fostering early interest is essential to sustain the physician workforce. This expansion has occurred alongside increasing national concerns regarding physician shortages, particularly in primary care, rural medicine, and underserved communities. Osteopathic physicians disproportionately enter these fields, underscoring the importance of strengthening the pipeline of future DO applicants to maintain workforce capacity [3,4]. Osteopathic physicians contribute a distinct perspective to US healthcare, and their expanding presence may help mitigate projected physician shortages. Early exposure during high school, when career choices are actively forming, can enhance awareness of osteopathic medicine as a potential pathway.

Experiential outreach programs have been shown to positively influence student motivation and clarity regarding health career pathways. Studies on “mini-medical schools” and immersion initiatives demonstrate that such exposure enhances interest in pursuing medicine [5-7]. For example, a program at the West Virginia School of Osteopathic Medicine showed that participation in a high school mini-medical school significantly improved students’ understanding of the physician’s role and increased their interest in pursuing a career in medicine [8]. On a similar note, the Med-Achieve program at Touro College of Osteopathic Medicine paired underrepresented minority high school students with medical student mentors, helping to reduce barriers such as limited guidance and financial constraints [8]. Pipeline programs have also been associated with greater student confidence, increased perceived feasibility of pursuing medicine, and strengthened intentions to apply to healthcare-related programs, particularly among students from historically excluded backgrounds [9,10].

While general medical outreach programs have proven valuable, there remains a need to expand them by providing early experiential exposure specifically to osteopathic medicine. Despite the success of existing initiatives, few have emphasized the unique philosophy and training that distinguish the osteopathic pathway. National awareness campaigns such as the American Osteopathic Association’s “The Doctor Will Hear You Now” initiative reflect growing efforts to enhance recognition of the DO profession, yet sustained exposure at the pre-college level remains limited [4]. The NextGenDO program was developed as a gamified, immersive event to provide high school students with direct interaction with osteopathic medical students and hands-on exposure to osteopathic principles. The objective of this study was to evaluate whether participation in the NextGenDO program improved high school students’ familiarity with osteopathic medicine, interest in pursuing a medical career, confidence in becoming a physician, and understanding of the pathway to medical school.

## Materials and methods

**Figure 1:**
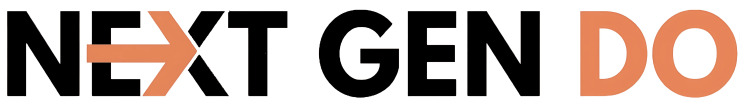
Logo of the NextGenDO program. Figure designed by Kenneth Quezada on Canva. Software: Canva Pro (Canva Inc., Perth, Australia).

This was a prospective, observational educational research study conducted to assess the impact of early exposure to Osteopathic Medicine on high school students through participation in the NextGenDO program. This event, held at the Orlando College of Osteopathic Medicine(Winter Garden, FL) was a single-day intervention designed for high school students considering careers in medicine (Figure 1). The program incorporated gamified learning stations, interactive demonstrations led by medical student volunteers, and structured discussions aimed at increasing understanding of osteopathic philosophy, medical school pathways, and careers in healthcare. Intervention content was delivered using standardized station guides, survey items followed a uniform Likert-scale format, and data handling procedures were applied consistently across all participants.

A total of 26 high school students (grades 9-12) enrolled in their schools’ medical track programs were eligible to participate in the event, with 23 (88.5%) completing the pre-survey and 21 (80.8%) completing the post-survey. Participation in the NextGenDO event was voluntary, and participation in the research component was optional, with no incentives or consequences for participation. This study was reviewed and deemed exempt by the institutional review board, in accordance with NIH guidelines for minimal risk studies (OCOM-E-2025-0016, Orlando College of Osteopathic Medicine, July 1, 2025). 

Survey instrument

Participants completed pre- and post-intervention surveys consisting of demographic items, prior exposure to osteopathic medicine, and Likert-scale questions assessing familiarity with osteopathic medicine, interest in becoming a physician, confidence in pursuing a medical career, and confidence in understanding the path to medical school. Survey questions were developed by the research team and reviewed for clarity and content validity by faculty members with experience in medical education.

Event structure

The program consisted of multiple structured stations, including: (1) an osteopathic manipulative treatment demonstration station; (2) a gamified anatomy and physiology challenge; (3) a medical school question and answer (Q&A) panel with Osteopathic Medical Student (OMS)-II student volunteers; and (4) a mock case-based learning (CBL) activity. Each station was designed to last 15-20 minutes, allowing small groups of students to rotate through all activities. Instruction was standardized using station guides to ensure consistent delivery across facilitators.

Data collection and statistical analysis

Survey data were collected electronically using Google Forms (Google LLC, Mountain View, CA). Responses were automatically compiled into a secure Google Sheets (Google LLC, Mountain View, CA) database and stored within the platform for analysis. The research team conducted manual verification within Google Sheets to ensure accuracy, completeness, and validity prior to statistical evaluation. Pre- and post-survey responses were paired using non-identifiable numerical codes assigned to each participant. Descriptive statistics (mean, median, mode) were calculated for all Likert-scale items. Because the data were ordinal and not normally distributed, the Wilcoxon signed-rank test was used to assess differences between pre- and post-intervention scores. A p-value of <0.05 was considered statistically significant. All analyses were conducted using R statistical software (R Foundation for Statistical Computing, Vienna, Austria).

## Results

**Figure 2:**
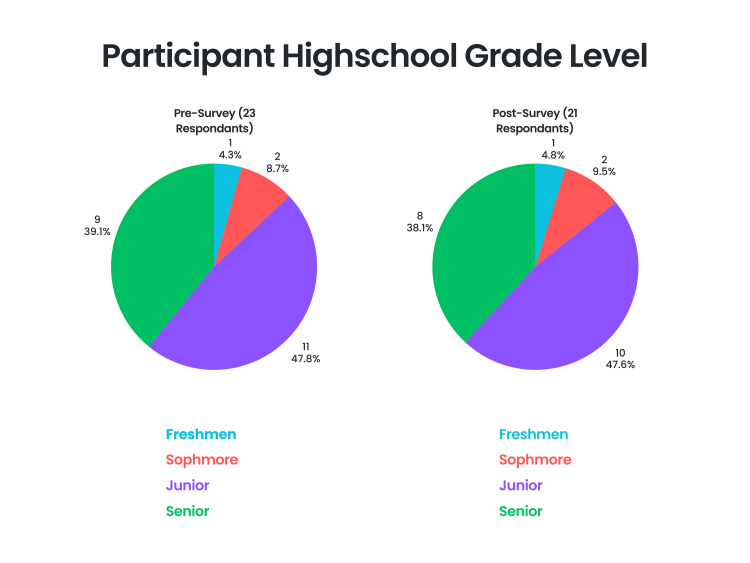
Distribution of participant high school grade level Figure designed by Ledio Gjunkshi using Canva Pro (Canva Inc., Perth, Australia).

Out of 26 eligible participants, only 23 (88.5%) completed the pre-survey and 21 (80.8%) completed the post-survey. Among the 23 participants who completed the pre-survey were nine high school seniors (39.1%), 11 high school juniors (47.8%), two high school sophomores (8.7%), and one high school freshman (4.3%). Among the 21 participants who completed the post-survey were eight high school seniors (38.1%), 10 high school juniors (47.6%), two high school sophomores (9.5%), and one high school freshman (4.8%) (Figure 2). 

Demographics

Out of the 23 pre-survey participants, 22 (95.7%) selected their gender as female, and one (4.3%) selected their gender as male. Regarding race/ethnicity, nine (39.1%) selected White, seven (30.4%) selected Hispanic/Latino, four (17.4%) selected Asian, two (8.7%) selected Black/African American, and one (4.3%) selected two or more races. Regarding first-generation college students, five (21.7%) selected that they would be a first-generation college student, and 18 (78.3%) selected that they would not be a first-generation college student. Regarding knowing physicians, 13 (56.5%) selected that they do know physicians personally, and 10 (43.5%) selected that they do not know any physicians personally. Regarding medical school, seven (30.4%) selected that someone in their family has attended medical school, and 16 (69.6%) selected that no one in their family has attended medical school.

**Figure 3:**
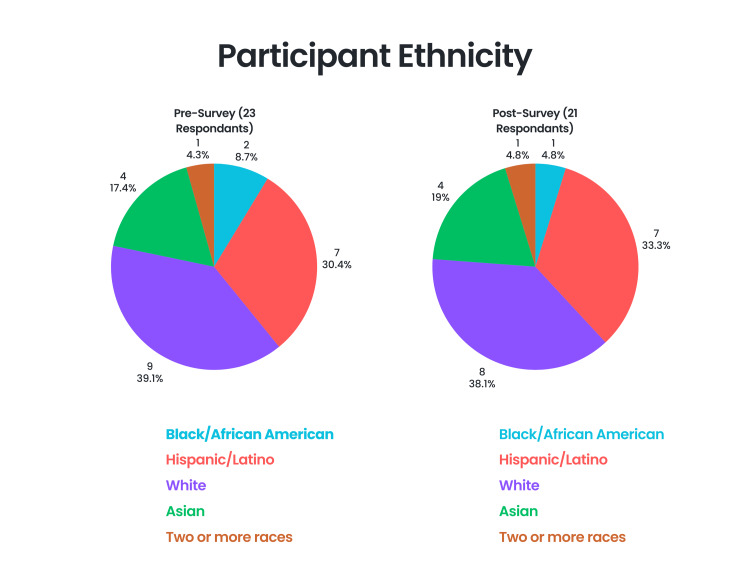
Distribution of participant race/ethnicity Figure designed by Ledio Gjunkshi using Canva Pro (Canva Inc., Perth, Australia).

**Figure 4:**
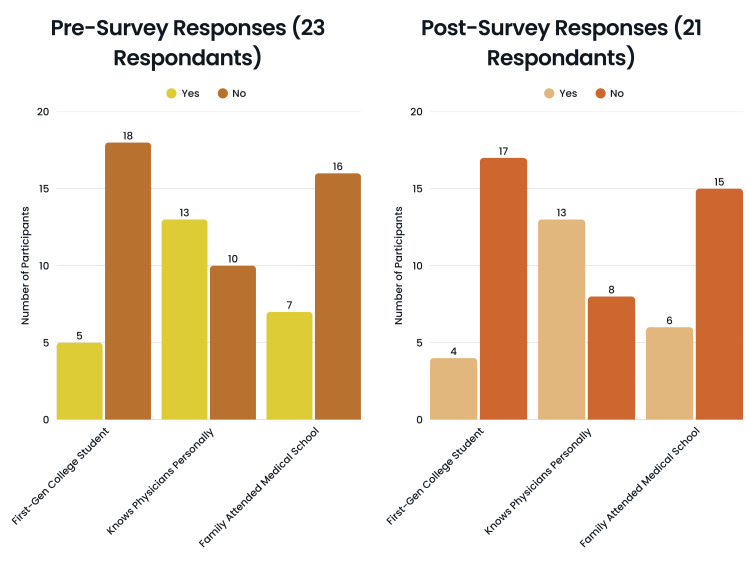
Participant responses regarding first-generation college status, personal acquaintance with physicians, and family history of medical school attendance Figure designed by Ledio Gjunkshi using Canva Pro (Canva Inc., Perth, Australia).

Out of the 21 post-survey participants, 20 (95.2%) selected their gender as female, and one (4.8%) selected their gender as male. Regarding race/ethnicity, eight (38.1%) selected White, seven (33.3%) selected Hispanic/Latino, 4 (19%) selected Asian, one (4.8%) selected Black/African American, and one (4.8%) selected two or more races (Figure 3). Regarding first-generation college students, four (19%) selected that they would be a first-generation college student, and 17 (81%) selected that they would not be a first-generation college student. Regarding knowing physicians, 13 (61.9%) selected that they do know physicians personally, and eight (38.1%) selected that they do not know any physicians personally. Regarding medical school, six (28.6%) selected that someone in their family has attended medical school, and 15 (71.4%) selected that no one in their family has attended medical school (Figure 4). 

Baseline knowledge and interest

Out of the 23 pre-survey participants, 19 (82.6%) selected that they heard of a Doctor of Osteopathic Medicine (DO), and four (17.4%) selected that they had not heard of a DO before (Figure 3). The remaining questions in this section were asked on a 5-point Likert scale and assigned values using the following variable in answer choices: “1 = Not”, “2 = Slightly”, “3 = Moderately”, “4 = Very”, “5 = Extremely”. Regarding familiarity with Osteopathic Medicine, two (8.7%) selected “Not at all familiar”, nine (39.1%) selected “Slightly familiar”, nine (39.1%) selected “Moderately familiar”, one (4.3%) selected “Very familiar”, and two (8.7%) selected “Extremely familiar”. Regarding interest in becoming a physician, two (8.7%) selected “Not interested”, four (17.4%) selected “Slightly interested”, four (17.4%) selected “Moderately interested”, five (21.7%) selected “Very interested”, and eight (34.8%) selected “Extremely interested”. Regarding the participants' confidence in pursuing a career in medicine, one (4.3%) selected “Slightly confident”, five (21.7%) selected “Moderately confident”, 11 (47.8%) selected “Very confident”, and six (26.1%) “Extremely confident”. Regarding the participants’ confidence in knowledge regarding the path to becoming a physician, one (4.3%) selected “Not at all confident”, 1 (4.3%) selected “Slightly confident”, 11 (47.8%) selected “Moderately confident”, six (26.1%) selected “Very confident”, and four (17.4%) selected “Extremely confident”.

Post knowledge and interest

**Figure 5:**
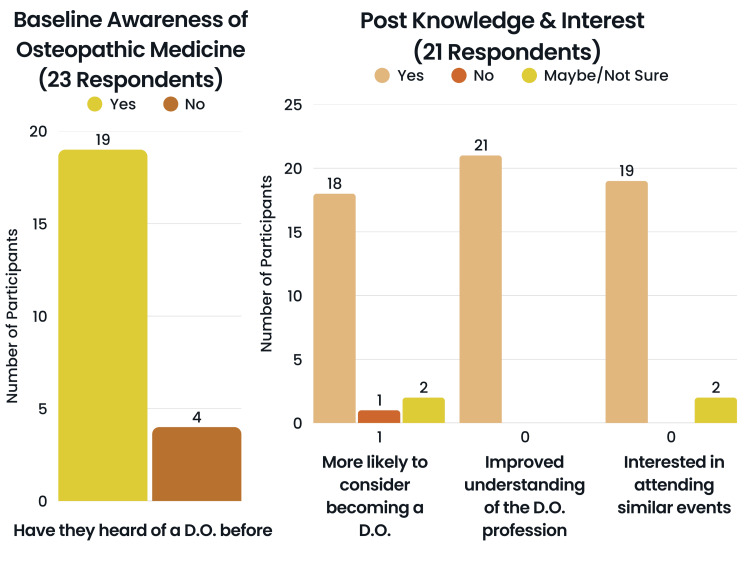
Pre- and post-survey responses of the participants The responses are regarding prior awareness of Doctors of Osteopathic Medicine (DOs), likelihood of considering a DO career, perceived improvement in understanding of the DO profession, interest in attending future events, and interest in shadowing a DO. Infographic designed by Ledio Gjunkshi using Canva Pro (Canva Inc., Perth, Australia).

Out of 21 post-survey participants, regarding more likely to consider becoming a DO post-event, 18 (85.7%) selected “Yes”, one (4.8%) selected “No” and two (9.5%) selected “Not sure”. Regarding understanding of a DO post-event, 21 (100%) selected that the event helped them better understand what a DO is. Regarding attending another event in the future, 19 (90.5%) selected “Yes”, two (9.5%) selected “Maybe”. Regarding participants’ interest in shadowing a DO or joining a health-related mentorship program, 19 (90.5%) selected “Yes”, and two (9.5%) selected “Maybe”. The remaining questions in this section were asked on a 5-point Likert scale and assigned values using the following variable in answer choices: “1 = Not”, “2 = Slightly”, “3 = Moderately”, “4 = Very”, “5 = Extremely”. Regarding familiarity with Osteopathic Medicine post-event, two (9.5%) selected “Moderately familiar”, 14 (66.7%) selected “Very familiar”, and five (23.8%) selected “Extremely familiar”. The Wilcoxon signed-rank test supported rejection of the null hypothesis (p-value = 0.00000602), therefore it was concluded that there was a significant increase in familiarity with Osteopathic Medicine. Regarding interest in becoming a physician post-event, one (4.8%) selected “Not interested”, two (9.5%) selected “Slightly interested”, five (23.8%) selected “Moderately interested”, two (9.5%) selected “Very interested”, and 11 (52.4%) selected “Extremely interested”. The Wilcoxon signed-rank test did not support the rejection of the null hypothesis (p-value = 0.31100000), therefore it was concluded that there was no significant increase in the participants' interest in medicine. Regarding the participants' confidence in pursuing a career in medicine post-event, three (14.3%) selected “Moderately confident”, 12 (57.1%) selected “Very confident”, and six (28.6%) “Extremely confident”. The Wilcoxon signed-rank test did not support the rejection of the null hypothesis (p-value = 0.49500000), therefore it was concluded that there was no significant increase in the participants' confidence to pursue a career in medicine. Regarding the participants’ confidence in knowledge regarding the path to becoming a physician post event, one (4.8%) selected “Not at all confident”, one (4.8%) selected “Slightly confident”, three (14.3%) selected “Moderately confident”, 10 (47.6%) selected “Very confident”, and six (28.6%) selected “Extremely confident” (Figure 5). The Wilcoxon signed-rank test did not support the rejection of the null hypothesis (p-value = 0.09120000), therefore while this value is approaching significance, it was concluded that there was no significant increase in the participants' confidence in knowledge regarding the path to becoming a physician (Table 1).

**Table 1:** Descriptive statistics and Wilcoxon signed-rank test results 1 Not, 2 Slightly, 3 Moderately, 4 Very, 5 Extremely

Metric	Familiarity with Osteopathic Medicine	Interest in Becoming a Physician	Confidence to Pursue a Career in Medicine	Confidence in Knowledge of Path to Becoming a Physician
Mean (Pre)	2.65	3.57	3.96	3.48
Mean (Post)	4.14	3.95	4.14	3.90
Median (Pre)	3	4	4	3
Median (Post)	4	5	4	4
Mode (Pre)	2	5	4	3
Mode (Post)	4	5	4	4
Wilcoxon Test Statistic (V)	55.0	200.0	214.5	172.5
p-value	0.0000602	0.311	0.495	0.0912

## Discussion

Analysis of pre- and post-survey data demonstrated that participants in the NextGenDO program gained greater understanding and familiarity with osteopathic medicine. Although increases were also observed in interest in becoming a physician, confidence in pursuing a medical career, and knowledge of the pathway to medical school, these differences did not reach statistical significance, likely due to confounding factors and the limited sample size. Post-event, most students indicated they were more likely to consider a DO career, expressed interest in shadowing osteopathic physicians, reported willingness to attend similar programs, and affirmed that the experience improved their comprehension of the osteopathic profession.

In the United States, approximately 11% of practicing physicians and 28% of medical students are Doctors of Osteopathic Medicine (DOs), with the remainder holding Doctor of Medicine (MD) degrees. Despite steady growth in the number of osteopathic schools and practitioners, continued advocacy and public education are necessary [8,11]. The quadrupling of osteopathic medical student enrollment over the past three decades underscores this expansion, yet DOs still represent only one-quarter of the national medical student population [12].

The American Osteopathic Association has advanced awareness through initiatives such as the “#DOProud” campaign, which spans social media, television, and print outlets [13]. Reinforcing these national efforts, early exposure programs offer an effective strategy for introducing aspiring pre-medical students to osteopathic medicine, highlighting it as a distinct and valuable pathway within the physician workforce. These findings reiterate those of prior research demonstrating that immersive pre-medical enrichment programs can enhance learners’ confidence, increase their awareness of medical career pathways, and improve their understanding of the physician’s role, further supporting the value of early experiential exposure in shaping students’ interest in osteopathic medicine [14,15].

Limitations

Several limitations should be considered when interpreting the findings of this study. Participation in the NextGenDO program and the associated surveys was voluntary, which may have introduced selection bias, as participants were primarily students already interested in pursuing medical careers and enrolled in medical track programs. Measurement validity is also a consideration, as outcomes were assessed using self-reported survey instruments that captured short-term perceptions rather than objective or longitudinal outcomes. In addition, the evaluation was limited to immediate post-event assessment, precluding conclusions regarding sustained impact on career interest, confidence, or educational trajectories. Finally, the small sample size limited statistical power and generalizability, underscoring the need for larger, multi-institutional studies with longer follow-up to more fully assess program effectiveness. While this study was not designed to evaluate long-term outcomes, early exposure initiatives such as NextGenDO may represent a potential strategy for increasing awareness of osteopathic medicine among pre-medical students. Future studies incorporating larger, more diverse cohorts and longitudinal follow-up are needed to determine whether early exposure programs influence application patterns, career decision-making, or contribute meaningfully to the physician pipeline.

## Conclusions

The NextGenDO program demonstrated an association with increased short-term familiarity with osteopathic medicine among participating high school students and was well received by attendees. Although improvements were observed in interest in medicine, confidence in pursuing a medical career, and understanding of the medical school pathway, these changes did not reach statistical significance and should be interpreted cautiously given the exploratory design, small sample size, and short-term outcome assessment. These findings suggest that early, structured exposure initiatives may support awareness of osteopathic medicine, but further studies with larger, more diverse cohorts and longitudinal follow-up are needed to better evaluate their sustained educational impact and generalizability.

## Appendices

**Table 2:** Pre-survey questions

Pre-Survey Question	Response Options
What grade are you in?	9th; 10th; 11th; 12th
What is your gender?	Male; Female; Prefer not to say
What is your race/ethnicity?	Black/African American; Hispanic/Latino; White; Asian; Native American or Alaska Native; Native Hawaiian or Other Pacific Islander; Two or more races; Prefer not to say
If you go to college, would you be a first-generation college student?	Yes; No; Not sure
Do you know any physicians personally?	Yes; No
Has anyone in your family attended medical school?	Yes; No
Have you ever heard of a Doctor of Osteopathic Medicine (DO)?	Yes; No; Not sure
How familiar are you with Osteopathic Medicine?	Not at all familiar; Slightly familiar; Moderately familiar; Very familiar; Extremely familiar
How interested are you in becoming a physician?	Not interested; Slightly interested; Moderately interested; Very interested; Extremely interested
How confident are you in your ability to pursue a career in medicine?	Not at all confident; Slightly confident; Moderately confident; Very confident; Extremely confident
How confident are you in your knowledge about the path to becoming a physician (college, MCAT, medical school, etc.)?	Not at all confident; Slightly confident; Moderately confident; Very confident; Extremely confident

**Table 3:** Post-survey questions

Post-Survey Question	Response Options
What grade are you in?	9th; 10th; 11th; 12th
What is your gender?	Male; Female; Prefer not to say
What is your race/ethnicity?	Black/African American; Hispanic/Latino; White; Asian; Native American or Alaska Native; Native Hawaiian or Other Pacific Islander; Two or more races; Prefer not to say
If you go to college, would you be a first-generation college student?	Yes; No; Not sure
Do you know any physicians personally?	Yes; No
Has anyone in your family attended medical school?	Yes; No
After today, how familiar are you with Osteopathic Medicine?	Not at all familiar; Slightly familiar; Moderately familiar; Very familiar; Extremely familiar
After today, how interested are you in becoming a physician?	Not interested; Slightly interested; Moderately interested; Very interested; Extremely interested
How confident are you in your ability to pursue a career in medicine?	Not at all confident; Slightly confident; Moderately confident; Very confident; Extremely confident
How confident are you in your knowledge about the path to becoming a physician (college, MCAT, medical school, etc.)?	Not at all confident; Slightly confident; Moderately confident; Very confident; Extremely confident
Are you now more likely to consider becoming a DO?	Yes; No; Not sure
Did this event help you better understand what a DO is?	Yes; No
Would you be interested in attending another event like this in the future?	Yes; No; Maybe
Would you be interested in shadowing a DO or joining a health-related mentorship program?	Yes; No; Maybe
